# AI-Driven Real-Time Phase Optimization for Energy Harvesting-Enabled Dual-IRS Cooperative NOMA Under Non-Line-of-Sight Conditions

**DOI:** 10.3390/s26030980

**Published:** 2026-02-03

**Authors:** Yasir Al-Ghafri, Hafiz M. Asif, Zia Nadir, Naser Tarhuni

**Affiliations:** 1 Department of Electrical and Computer Engineering, Sultan Qaboos University (SQU), Muscat 123, Oman; s132332@student.squ.edu.om (Y.A.-G.); tarhuni@squ.edu.om (N.T.); 2 Affiliated with UNESCO Chair on AI, Communications and Information Research Center (CIRC)-Sultan Qaboos University (SQU), Muscat 123, Oman

**Keywords:** energy harvesting (EH), intelligent reflecting surfaces (IRS), non-line-of-sight (NLoS), non-orthogonal multiple access (NOMA)

## Abstract

In this paper, a wireless network architecture is considered that combines double intelligent reflecting surfaces (IRSs), energy harvesting (EH), and non-orthogonal multiple access (NOMA) with cooperative relaying (C-NOMA) to leverage the performance of non-line-of-sight (NLoS) communication mainly and incorporate energy efficiency in next-generation networks. To optimize the phase shifts of both IRSs, we employ a machine learning model that offers a low-complexity alternative to traditional optimization methods. This lightweight learning-based approach is introduced to predict effective IRS phase shift configurations without relying on solver-generated labels or repeated iterations. The model learns from channel behavior and system observations, which allows it to react rapidly under dynamic channel conditions. Numerical analysis demonstrates the validity of the proposed architecture in providing considerable improvements in spectral efficiency and service reliability through the integration of energy harvesting and relay-based communication compared with conventional systems, thereby facilitating green communication systems.

## 1. Introduction

Since the first generation (1G), several access techniques have evolved to support multiple users, with a focus on improving spectrum utilization. These include frequency division multiple access (FDMA), time division multiple access (TDMA), code division multiple access (CDMA), and orthogonal frequency division multiple access (OFDMA), and they were adopted early on across generations 1G, 2G, 3G, and 4G [1]. These methods, known as orthogonal multiple access (OMA), separate users by time, frequency, or code to prevent interference. OMA enables low-cost, low-complexity receiver designs; however, its dependence on orthogonal resources limits user bandwidth [2]. As an alternative to OMA, NOMA was introduced, which allows for a greater number of users compared with OMA by increasing the receiver complexity to handle overlapping signals, making it highly suitable for Internet of Things (IoT), massive machine-type communications (mMTC), and smart city infrastructures [3]. Intelligent reflecting surfaces (IRSs) further add a dynamic dimension to NOMA, enabling electromagnetic wave redirection to overcome signal blockages and thereby enhance link quality in non-line-of-sight (NLoS) environments. IRS technology offers a flexible and convenient solution for improving wireless communication, as IRS components can be easily deployed on facades, walls, and ceilings with incredibly low power consumption [4]. Remarkably, the IRS does not require an active RF component in its technology to process and retransmit the signal, which provides an energy-efficient solution implicitly. Structurally, the IRS is made up of three essential layers:

- Metasurface Layer: This is the top layer fabricated with sub-wavelength-sized particles in a planar array for electromagnetic wave modulation.
- Copper Layer: This is a layer below the metasurface that prevents the leakage of signal energy and keeps the signals within the desired paths.
- Circuit Board and IRS Controller: The main circuit board is connected to the IRS controller, which is the controller that is in charge of the adaptability of the surface.

However, IRS performance heavily relies on the availability and quality of the natural signal paths, which could be seriously degraded under NLoS scenarios due to interference from obstacles that attenuate signal strength.

Similarly, EH-NOMA introduces a new level of robustness in wireless networks, particularly in challenging NLoS environments, where conventional power supplies are scarce or unavailable. This capability provides relief from not only the burden of power infrastructure but also incessant and reliable network performance; hence, EH-NOMA becomes quite promising for remote areas and IoT applications, which require day-to-day network uptime [5,6]. It enables devices to operate independently of conventional power grids, allowing for more resilient, scalable, and energy-efficient network deployments.

In such scenarios where NLoS communication dominates, the addition of IRSs to EH-NOMA systems introduces a revolutionary method for mitigating the NLoS limitation and amplifying the core of NOMA [7]. The IRS dynamically reconfigures the wireless propagation environment by establishing virtual LoS alternatives and compensates for performance degradation caused by blockages.

Nevertheless, phase-shift optimization across multiple IRSs in real time remains a challenging, non-convex task, particularly under joint energy and time allocation constraints. Standard optimization approaches (e.g., semidefinite programming (SDP) and alternating optimization) provide superior performance but are not readily applicable to real-time or large-scale scenarios due to their iterative nature and computational demands. To address these scalability limitations, machine learning (ML) and,. in particular, deep learning have recently gained attention as alternative approaches.

While a limited number of studies have explored the use of machine learning in order to optimize phase shifts in multiple-IRS systems, their work mainly focused on conventional MISO beamforming scenarios using neural networks. These approaches were designed to minimize transmit power or improve active beamforming without addressing the complexities introduced by more advanced wireless set-ups. Our work, by contrast, examines a fundamentally different system architecture (i.e., a wireless-powered cooperative NOMA network supported by dual IRSs). While NOMA remains a promising technology that researchers have recently focused on, several fundamental challenges remain when integrating this architecture with dual-IRS systems, energy harvesting, and cooperative relaying under NLoS conditions. Current studies primarily focus on (1) allocation using a single IRS beam or (2) passive IRS–NOMA rate optimization. However, the following multidimensional challenges remain:

(1) To date, the joint impact of EH, dual IRS, and cooperative NOMA has not been modeled in ML-based optimization at the time of this publication.

(2) In addition, there is no prior work that learns directly from the wireless environment without supervised labels from convex optimization (CVX) or SDP at the time of this publication. Hence, the main contributions of the study are as follows:

- NLoS Mitigation via IRS: This work proposes a novel system using a double IRS which can dynamically reconfigure the propagation environment by effectively mitigating NLoS issues and thus providing robust communication links.
- Machine Learning-Based IRS Optimization: Instead of solving complex optimization problems for IRS phase shift optimization, we introduce a machine learning-based approach to predict optimal phase shifts and reduce computational complexity.
- Energy Harvesting in NOMA: This energy harvesting system uses power to embed energy harvesting into the cooperative NOMA system for energy sustainability in IoT devices.

The remainder of this paper is structured as follows. Section 2 presents related work on NOMA and other advanced solutions to manage non-line-of-sight (NLoS) transmission challenges, along with their respective evaluation methodologies, performance benchmarks, shifts, and key findings. Section 3 describes the structure of the artificial neural network (ANN) model used for optimization. Section 4 provides a comprehensive overview of the system model’s details, highlighting its components and assumptions. Section 5 presents the numerical results, analyzes the study’s findings, and provides relevant insights. Finally, Section 6 concludes the current research work.

## 2. Related Work

In this section, the existing work on mitigating the challenges of NLoS environments is presented. We primarily focus on studies of IRS-NOMA, EH-NOMA, and C-NOMA in this regard.

### 2.1. IRS-NOMA

The IRS’s involvement in wireless networks has brought about a significant change in how non-line-of-sight (NLoS) problems are addressed. By altering how wireless signals propagate through the environment, the IRS enables precise control of scattered signals. This ensures that reflected signals reach the intended users and reduces the interference caused by others. This allows an increase in energy efficiency and spectrum use [8].

The IRS makes the traditional NOMA system even more customizable when paired with NOMA. In contrast to conventional NOMA, which relies on fixed channel settings based on the signal environment, IRS-NOMA uses dynamic phase adjustment. These changes result in different user signal strengths, making NOMA more effective in challenging NLoS conditions. This combination helps IRS-NOMA blend into its surroundings and create stronger, more user-friendly communication [9].

In NLoS cases, where Rayleigh and Nakagami-m paths are employed, the IRS enhances system performance by creating virtual paths, analogous to LoS links. For example, one study [10] showed that IRSs combined with NOMA outperform traditional IRS-OMA in networks with high interference under Nakagami-m fading, yielding 20–30% higher data rates and fewer connection drops. However, existing IRS and IRS–radar studies [8,9,10] typically focus on simplified single-surface set-ups and offline optimization, and they do not consider energy harvesting or cooperative NOMA operation under real-time constraints, which motivates the AI-driven dual-IRS C-NOMA framework proposed in this work.

### 2.2. Energy Harvesting-Enabled NOMA

Energy harvesting is a viable option in NLoS environments where obstacles such as buildings or terrain impede signal strength and cause power loss. Devices with energy harvesting technology can control surrounding energy sources and ensure waste-free operation even in areas where traditional methods of transmission and power are unavailable [11]. These systems enable effective communication over long distances and overcome physical obstacles.

Although energy consumption alone cannot fully solve the NLoS communication problem, integration with the NOMA system increases productivity and guarantees stable and portable operation with a wide range of applications. This connection helps address challenges such as fading and interference while also extending the network’s operating life. Studies examining Nakagami-m and Rayleigh fading channels, which characterize NLoS conditions, consistently highlight the superior performance of EH-NOMA systems over conventional orthogonal multiple access (OMA) methods [12].

Recent works have investigated energy harvesting-enabled NOMA under different architectures. In [13], a MIMO NOMA system with nonlinear energy harvesting and imperfect CSI has been analyzed. The authors derive explicit expressions for the throughput and outage of an SWIPT-based relay node that harvests and forwards data to a distant user. However, their model remains a single-relay downlink without intelligent reflecting surfaces or cooperative NOMA relaying across multiple hops, in which they focus on analytical performance evaluation rather than real-time optimization.

On the other hand, the authors of [14] considered secure full-duplex NOMA with energy harvesting, in which an energy transmitter both powers the NOMA node and jams an eavesdropper, and they derived secrecy and reliability metrics under CSI, hardware, and SIC imperfections. Yet, this work still assumed a single-cell setting with conventional RF links and no IRS-assisted NLoS mitigation or learning-based control. In the IoT context, the study performed in [15] proposed an energy-harvesting buffer-aided cooperative NOMA network with relay selection based on the joint state of data and energy buffers to improve outage probability and throughput. Nevertheless, this approach captures random energy arrivals and cooperative relaying; it relies on conventional half-duplex relays without IRSs. In addition, they used simple linear EH models and did not consider joint phase shifts or beamforming optimization. Hence, existing EH-NOMA studies either focus on analytical performance or relay or parameter selection in conventional RF networks, but none address dual-IRS-assisted, EH-enabled cooperative NOMA with low-complexity AI-driven real-time phase optimization under NLoS conditions, which is the focus of this work.

### 2.3. Cooperative NOMA

Researchers have explored integrating cooperative communications technology with NOMA to mitigate wireless channel fading and enhance wireless coverage. A cooperative NOMA is one in which nearby users act as relays. A method for transmitting data to more distant users is proposed in [16]. Similarly, refs. [17,18] investigated cooperative NOMA systems in decode-and-forward (DF) and amplify-and-forward (AF) methods. They studied the system’s outage performance and bit error rate under these configurations. Moving beyond half-duplex (HD) systems, full-duplex (FD) NOMA has emerged as a promising approach. FD enables simultaneous transmission and reception, thereby improving spectral efficiency [19]. Other studies [20,21,22] showed that FD cooperative NOMA achieves a 32% higher spectral efficiency than half-duplex (HD) set-ups. They also studied loop self-interference (LSI) and its impact on system performance.

Research on cooperative NOMA systems has expanded beyond user-assisted relaying to include set-ups with dedicated relays. Studies indicate that systems using amplify-and-forward (AF) relays outperform orthogonal multiple access (OMA) in terms of coding gain, outage performance, and throughput. Under Rayleigh fading, AF-based C-NOMA systems achieve 25% higher throughput than OMA, whereas DF systems reduce the outage probability by 35%, as noted in [23]. While initial research focused on Rayleigh fading channels, some studies [24,25] extended these to Nakagami-m-fading channels and analyzed the outage probability and sum rate under this more comprehensive model, which exhibits adaptation to various fading conditions. However, all of these previous studies still considered conventional cooperative NOMA with fixed relay links and no dual-IRS assistance. Plus, they lack any adaptation of a low-complexity AI scheme for real-time phase shift control in NLoS channels, which is exactly the scenario addressed in this work.

## 3. System Model

**Figure 1:**
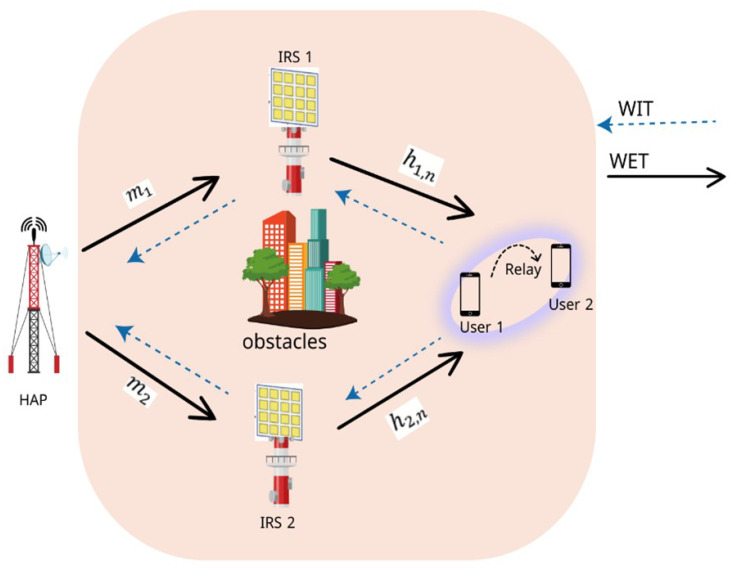
System model of the proposed energy harvesting-assisted, dual IRS-enabled C-NOMA system.

Figure 1 shows the block-level components of the proposed system. The system model incorporates advanced techniques, including NOMA for efficient resource allocation, IRS for signal propagation enhancement, and machine learning for data-driven performance predictions. Specifically, the system employs a double-IRS arrangement to mitigate NLoS issues caused by obstacles that act as passive signal relays, reflecting RF signals toward the intended users to establish reliable communication links. The presence of two IRSs significantly enhances the system’s ability to maintain communication when direct paths are unavailable. On the user side of Figure 1, the architecture incorporates a signal and power splitting mechanism for both users, $U_{1}$ and $U_{2}$, to harvest energy and process information simultaneously. We examine a wireless system that includes two intelligent reflecting surfaces (IRSs), designated IRS1 and IRS2, a hybrid access point (HAP), a near user, and a far user. $N_{1}$ and $N_{2}$ denote the number of reflecting elements in IRS1 and IRS2, respectively, and both the HAP and the users are assumed to have single-antenna configurations for simplicity. Both wireless energy transfer (WET) and wireless information transmission (WIT) must rely on IRSs, as a physical barrier prevents direct contact between the HAP and the users.

The near user’s cooperative relaying helps the remote user receive a stronger signal, further improving signal reception. There are two separate stages to the transmission process:

1. Energy Transfer Phase ($t_{0}$): The HAP broadcasts energy with power $P_{0}$ to the users.
2. Information Transmission Phase ($t_{1}$): The users send their data back to the HAP.

We assume that $t_{0}+t_{1}=1$ in a general manner. The network layout is designed to mitigate signal propagation challenges arising from obstacles or deep fading in non-line-of-sight (NLoS) environments. The components of the network and their configurations are as follows:

- HAP: Located at (−20,0), the HAP serves as the primary source of both energy and information. It employs a single antenna and operates in a time division manner to support the WET and WIT phases.
- IRS_1_ and IRS_2_: The two IRSs are positioned at (0,30) and (0,−30), respectively. Each IRS is equipped with $N_{1}=N_{2}=50$ reflecting elements. These surfaces dynamically adjust their phase shifts to enhance signal strength and effectively manage interference.
- Users: Users are randomly distributed within a square region centered at (20,0) with dimensions of 30m×30m, reflecting typical user mobility patterns and varying channel conditions.

### 3.1. Statistical Modeling

The channel gains from the HAP to ${\mathrm{IRS}}_{1}$ and ${\mathrm{IRS}}_{2}$ are denoted by $m_{1}\in C^{1\times N_{1}}$ and $m_{2}\in C^{1\times N_{2}}$, respectively. Similarly, the channels between ${\mathrm{IRS}}_{1}$ and the two users are represented by $h_{1,1}\in C^{N_{1}\times 1}$ and $h_{1,2}\in C^{N_{1}\times 1}$. Furthermore, the channels between ${\mathrm{IRS}}_{2}$ and the near user and between ${\mathrm{IRS}}_{2}$ and the far user are denoted by $h_{2,1}\in C^{N_{2}\times 1}$ and $h_{2,2}\in C^{N_{2}\times 1}$, respectively.

We consider a severe NLoS scenario where the direct HAP-to-user links are fully blocked by obstacles; therefore, the direct channel is neglected, i.e., $h_{\mathrm{direct}}=0$. The IRS-assisted links may still include dominant specular components, depending on the deployment geometry. Hence, the HAP–IRS and IRS–user channels are modeled using Rician fading with distance-dependent path loss. All channels are assumed to be quasi-static within each transmission block, which is suitable for low-mobility users and fixed IRS deployment. We assume unit-modulus reflection for the IRS elements, while phase alignment is treated as an ideal baseline. The impact of CSI imperfections is discussed later.

The channel coefficient for a link at distance *d* is modeled as follows:

(1) $$h=\sqrt{PL(d)}\;\tilde{h},$$

where PL(d) denotes the large-scale path loss attenuation and $\tilde{h}$ captures small-scale fading. In this work, $\tilde{h}$ follows a Rician distribution for the IRS-assisted links to account for a possible LoS component.

Considering that the IRS-assisted links may contain LoS components, the HAP–IRS and IRS–user channels are modeled using Rician fading with distinct Rician K factors for different segments. Specifically, the HAP–IRS links are expressed as follows:

(2) $$m_{i}=\sqrt{\frac{K_{HI}}{K_{HI}+1}}\;m_{i}^{\mathrm{LoS}}+\sqrt{\frac{1}{K_{HI}+1}}\;m_{i}^{\mathrm{NLoS}},\;i\in \left\{1,2\right\},$$

and the IRS–user links are expressed by

(3) $$h_{i,k}=\sqrt{\frac{K_{IU}}{K_{IU}+1}}\;h_{i,k}^{\mathrm{LoS}}+\sqrt{\frac{1}{K_{IU}+1}}\;h_{i,k}^{\mathrm{NLoS}},\;i\in \left\{1,2\right\},\;k\in \left\{1,2\right\},$$

where $K_{HI}$ and $K_{IU}$ denote the Rician K factors of the HAP–IRS and IRS–user links, respectively. The NLoS components follow circularly symmetric complex Gaussian distributions, namely $m_{i}^{\mathrm{NLoS}}∼\mathrm{CN}\left(0,I\right)$ and $h_{i,k}^{\mathrm{NLoS}}∼\mathrm{CN}\left(0,I\right)$. Unless otherwise stated, we set $K_{HI}=8\;\mathrm{dB}$ and $K_{IU}=3\;\mathrm{dB}$ to reflect stronger LoS conditions for the HAP–IRS links compared with the IRS–user links.

The effective channel gain $h_{e,k}$ is given by

(4) $$h_{e,k}=h_{\mathrm{direct},k}+h_{\mathrm{ref},k},$$

where $h_{\mathrm{direct},k}=0$ due to the considered severe blockage and $h_{\mathrm{ref},k}$ denotes the aggregate reflected contribution from both IRSs. The reflected contributions from both IRSs are expressed by

(5) $$h_{\mathrm{ref}}=\frac{1}{\sqrt{N}}\left(\sum_{n=1}^{N_{1}}e^{j\theta_{1,n}}h_{n,{\mathrm{IRS}}_{1}}+\sum_{n=1}^{N_{2}}e^{j\theta_{2,n}}h_{n,{\mathrm{IRS}}_{2}}\right),$$

(6) $$h_{\mathrm{ref},k}=\frac{1}{\sqrt{N}}\left(\sum_{n=1}^{N_{1}}e^{j\theta_{1,n}}h_{n,{\mathrm{IRS}}_{1},k}+\sum_{n=1}^{N_{2}}e^{j\theta_{2,n}}h_{n,{\mathrm{IRS}}_{2},k}\right)$$

where $N=N_{1}+N_{2}$ is the total number of reflecting elements across both IRSs and $h_{n,{\mathrm{IRS}}_{1}}$ and $h_{n,{\mathrm{IRS}}_{2}}$ denote the channel gains associated with the *n*th reflecting element of ${\mathrm{IRS}}_{1}$ and ${\mathrm{IRS}}_{2}$, respectively. The terms $\theta_{1,n}$ and $\theta_{2,n}$ represent the phase shifts applied by the corresponding IRS elements. Here, $h_{n,{\mathrm{IRS}}_{1}}$ (and $h_{n,{\mathrm{IRS}}_{2}}$) represents the cascaded channel coefficient associated with the *n*th element, which includes both the HAP–IRS and IRS–user links.

The scaling by $\sqrt{N}$ assumes that the IRS elements introduce constructive interference. This requires precise phase alignment, namely $e^{j\theta_{1,n}}$ and $e^{j\theta_{2,n}}$, for the IRS elements. However, perfect alignment may not always be achievable due to hardware imperfections or channel estimation errors.

#### 3.1.1. Imperfect CSI and Mobility Discussion

In the formulation discussed in Section 3.1, the IRS phase shifts are designed based on channel state information (CSI). While perfect CSI is often adopted to establish an upper bound, practical systems inevitably operate with imperfect CSI due to estimation noise, quantization, and feedback delay. To capture this effect, we consider a standard imperfect CSI model where the available channel estimates are given by

(7) $${\hat{m}}_{i}=m_{i}+\Delta m_{i},\;{\hat{h}}_{i,k}=h_{i,k}+\Delta h_{i,k},$$

where $\Delta m_{i}$ and $\Delta h_{i,k}$ represent the estimation errors, modeled as $\Delta m_{i}$∼$\mathrm{CN}(0,\sigma_{m}^{2}I)$ and $\Delta h_{i,k}$∼$\mathrm{CN}(0,\sigma_{h}^{2}I)$, respectively. Moreover, under user mobility, the channel may vary between estimation and IRS reconfiguration, which can be interpreted as channel aging. One commonly used model is

(8) $$g\left(t+\tau \right)=\rho \;g\left(t\right)+\sqrt{1-\rho^{2}}\;w,$$

where 0≤ρ≤1 captures temporal correlation (smaller ρ indicates higher mobility or larger feedback delay) and w∼CN(0,I). These effects reduce the phase alignment accuracy, thereby degrading the achievable rate and harvested energy. However, the proposed ML-based approach remains attractive because it provides fast inference once trained and enables more frequent IRS phase updates than iterative optimization methods.

#### 3.1.2. Performance Metrics

The SIC is applied to first decode and subtract the far user’s signal, enabling clean decoding of the near user’s signal. The far user, being located farther away, decodes its own signal while treating the near user’s signal as interference. For each user, k∈{near,far}.

The SINR for the near and far users is expressed as follows:

(9) $${\mathrm{SINR}}_{\mathrm{near}}=\left(\frac{\alpha_{\mathrm{near}}P_{t}{\left|h_{e,\mathrm{near}}\right|}^{2}}{\sigma^{2}}\right),$$

(10) $${\mathrm{SINR}}_{\mathrm{far}}=\left(\frac{\alpha_{\mathrm{far}}P_{t}{\left|h_{e,\mathrm{far}}\right|}^{2}}{\alpha_{\mathrm{near}}P_{t}{\left|h_{e,\mathrm{far}}\right|}^{2}+\sigma^{2}}\right),$$

where $\sigma^{2}$ denotes the noise power. The SIC mechanism ensures that the near user signal is decoded first, while the far user signal is treated as interference. The transmit power $P_{t}$ is dynamically computed as $P_{t}=\mathrm{SNR}\times \sigma^{2}$. The terms $|h_{e,\mathrm{near}}{|}^{2}$ and $|h_{e,\mathrm{far}}{|}^{2}$ denote the effective channel gains for the near and far users, respectively, considering both direct and IRS-reflected paths. The power-allocation factors are set to $\alpha_{\mathrm{near}}=0.4$ and $\alpha_{\mathrm{far}}=0.6$.

The harvested energy $E_{\mathrm{harvested}}$, measured in joules (or millijoules for practical applications), is given by

(11) $$E_{\mathrm{harvested}}=\eta P_{t}{\left|h_{e}\right|}^{2}t,$$

where η represents the energy harvesting efficiency and *t* is the allocated harvesting duration. The corresponding achievable rate is expressed as follows:

(12) $$R_{MM}=\log_{2}\left(1+\frac{P_{t}{\left|h_{e}\right|}^{2}}{\sigma^{2}}\right),$$

where $R_{MM}$ denotes the communication rate in bits per second per hertz (bps/Hz).

Outage probability is a critical metric that characterizes system reliability. For a threshold $\gamma_{\mathrm{th}}$, the outage probabilities for the near and far users are defined as follows:

(13) $$P_{\mathrm{out},\mathrm{near}}=\mathrm{Pr}\left({\mathrm{SINR}}_{\mathrm{near}}<\gamma_{\mathrm{th},\mathrm{near}}\right),$$

(14) $$P_{\mathrm{out},\mathrm{far}}=\mathrm{Pr}\left({\mathrm{SINR}}_{\mathrm{far}}<\gamma_{\mathrm{th},\mathrm{far}}\right).$$

The above expressions summarize the main performance metrics considered in this work, namely the harvested energy, the achievable rate, and the outage probabilities of the near and far users. Together, they capture the fundamental trade-off between energy harvesting time, transmit power, and link reliability in the proposed EH-enabled C-NOMA system. In the next subsection, we specify the system parameters and describe how these metrics are jointly improved using the proposed ML-based optimization framework.

### 3.2. System Parameters

The primary objective of the proposed system is to enhance both the achievable data rate and the amount of harvested energy by training the model to learn the most effective IRS phase configurations. The system should adapt to varying wireless conditions to improve overall system performance. Such a learning-based approach enhances the model’s reliability and adaptability in dynamic environments.

**Figure 2:**
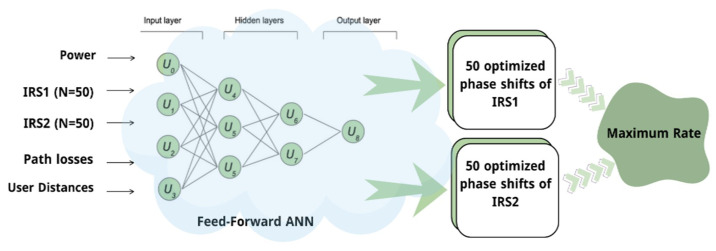
DNN-based optimization mechanism for IRS phase shifts.

Figure 2 shows a generic view of our optimization parameters. As shown in the figure, several comprehensive hyperparameter optimizations were conducted. This can be achieved by using grid search to ensure that the model is robust and stable across combinations of learning rates, batch sizes, dropout levels, and regularization terms. As shown in Figure 2, the final model architecture was carefully chosen based on the computational complexity and whether the model was able to generalize well to unseen data. Furthermore, we monitored both the training and validation loss curves over successive epochs to avoid overfitting.

To avoid training instability, we introduce a randomness factor of 0.3. This ensures that the generated phase shifts vary by no more than 30% of the full range. For convenience, we set the number of users $U_{n}=2$ and the noise spectral density $N_{0}=-154\;\mathrm{dBm}/\mathrm{Hz}$ to ensure a fair and direct comparison between our ML-based method and the benchmark SDP-based approach [7].

Increasing the number of users would require different optimization techniques and advanced machine learning models to handle IRS phase shifts, EH relay selection, and interference management efficiently, which is beyond the scope of this study. Other system settings are shown in Table 1.

## 4. ML-Based Optimization Approach

### 4.1. Selection of ML Framework

**Figure 3:**
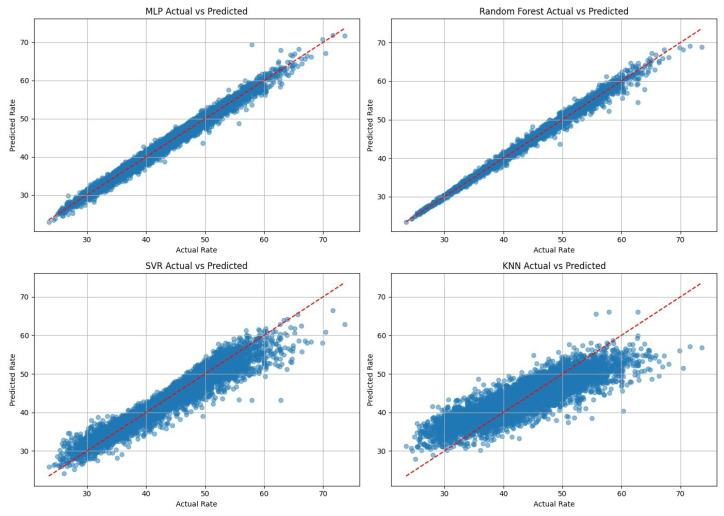
Comparing the prediction performance between different ML models.

Since jointly optimizing a large number of phase shifts in a dual-IRS system is both computationally demanding and resource-intensive, we conducted a comprehensive evaluation of several algorithms to identify the most effective machine learning approach for our system. Among these algorithms are the multi-layer perceptron (MLP), a type of deep neural network (DNN), random forest, support vector regression (SVR), and k-nearest neighbor (KNN). The comparison, presented in Figure 3, shows each model’s ability to predict the average rate values, and their peak performance metrics are summarized in Table 2 and Table 3. Although random forest delivered slightly higher predictive accuracy, mainly for rate prediction (scalar output), the primary objective of this work is real-time IRS phase optimization. These tasks require repeated inference under strict latency and memory constraints and involve learning nonlinear coupling among high-dimensional phase-related features.

An MLP was selected because it provides a compact parametric mapping with smooth, continuous outputs and constant-time inference per sample once trained. This property is incredibly important for practical IRS control, where phase decisions must be updated instantaneously, and the system is reliable under channel variations. The RF inference time, on the other hand, scales with the number of trees and their depth, which becomes less attractive for real-time deployment as the model size increases. Furthermore, we selected the multilayer perceptron (DNN) due to its proven ability to model complex nonlinear relationships [26], its architectural flexibility, and its suitability for real-time, AI-driven IRS-based wireless systems [27].

Also, one of our key considerations in this choice was inference efficiency. Specifically, MLPs, once trained, require minimal computational overhead during prediction, making them ideal for latency-sensitive or power-constrained environments. In contrast, random forest, despite its accuracy, relies on an ensemble of decision trees, which increases the prediction time in proportion to the number of estimators. This makes them less favorable for applications where fast, scalable, and energy-efficient performance is essential [28]. Reinforcement learning could also be considered for IRS control, particularly in interactive or long-horizon sequential environments. However, in our considered quasi-static block fading model, each channel realization is independent, and the phase design decision is made once per block. Specifically, the learning model we adopted is trained to approximate the mapping from input features to the desired performance output (equivalently, to an optimized phase design decision), which can be formulated as a regression task. RL, on the other hand, would require repeated online interaction with the environment, which increases computational and training overhead and may lead to unstable convergence.

The MLP network used has several layers, starting with 256 neurons, then 128, and finally 64. This structure was found to provide a favorable balance between prediction accuracy and model complexity, which is important for real-time IRS phase adaptation. In particular, larger networks yielded only marginal accuracy gains at the expense of higher inference costs, whereas smaller networks exhibited noticeable performance degradation. Moreover, rectified linear unit (ReLU) activation functions are used in our machine learning approach, as they work well for this type of optimization by introducing nonlinearity into the neural network. This enables the system to model complex relationships between inputs and outputs.

To ensure proper training and avoid overfitting, batch normalization is applied, and dropout layers with a rate of 0.4 are included. Additionally, L2 regularization with a factor of 0.01 is used to discourage the model from assigning excessive importance to any single feature or noise in the data.

After justifying the choice of learning model, the following subsection explains how this network is incorporated into the proposed optimization framework, including dataset creation, feature design, and the training procedure used to learn the mapping from system parameters to near-ideal IRS phase configurations.

### 4.2. DNN Optimization Mechanism

#### 4.2.1. ML Dataset and Feature Design

We generated a training dataset by simulating thousands of random IRS phase shift combinations, evaluating their performance, and selecting the best-performing configuration (i.e., with the highest rate or energy) for each scenario. The goal is to learn a function that maps a set of system inputs to their corresponding maximum achievable performance. The objective function can be expressed as follows:

(15) $$\max_{\left\{\theta_{n}\right\}}\left[\Gamma \;R_{MM}\left(\theta_{n}\right)+\left(1-\Gamma \right)\;E_{\mathrm{harvested}}\left(\theta_{n}\right)\right],$$

where $\theta_{n}$ are the IRS phase shifts for both ${\mathrm{IRS}}_{1}$ and ${\mathrm{IRS}}_{2}$, $R_{MM}$ is the achievable rate, $E_{\mathrm{harvested}}$ is the harvested energy, and Γ∈[0,1] is a weighting factor balancing the two objectives. The DNN learns to directly optimize phase shift vectors by predicting the IRS phase shift configurations that yield the best performance (rate or energy) given the system parameters. It can be modeled by

(16) $$f_{\mathrm{DNN}}:x^{\left(i\right)}\to {\hat{y}}^{\left(i\right)},$$

where $x^{\left(i\right)}$ is the input feature vector and ${\hat{y}}^{\left(i\right)}$ is the predicted performance metric (e.g., maximum rate or energy). The output layer of the DNN represents the phase shift vector $\theta =[\theta_{1},\theta_{2},\ldots ,\theta_{N}]$, where each $\theta_{i}\in \left[\pi ,-\pi \right]$. Furthermore, the model is trained using the mean squared error (MSE) loss function, which is given by

(17) $$L_{\mathrm{MSE}}=\frac{1}{n}\sum_{i=1}^{n}{\left({\hat{y}}^{\left(i\right)}-y_{\mathrm{true}}^{\left(i\right)}\right)}^{2},$$

where $y_{\mathrm{true}}^{\left(i\right)}$ denotes the best observed performance (rate or energy) for the ith scenario. We approached the optimization of IRS phase shifts as a supervised regression problem. To support this, we created a dataset of 10,000 samples, where each sample represents a unique set of system conditions, including the transmit power, path losses, distances, and randomly assigned IRS phase shifts, along with the resulting achievable rate. We chose these particular features as they play an important role in the signal strength shaping and overall performance in IRS-assisted NOMA systems.

**Figure 4:**
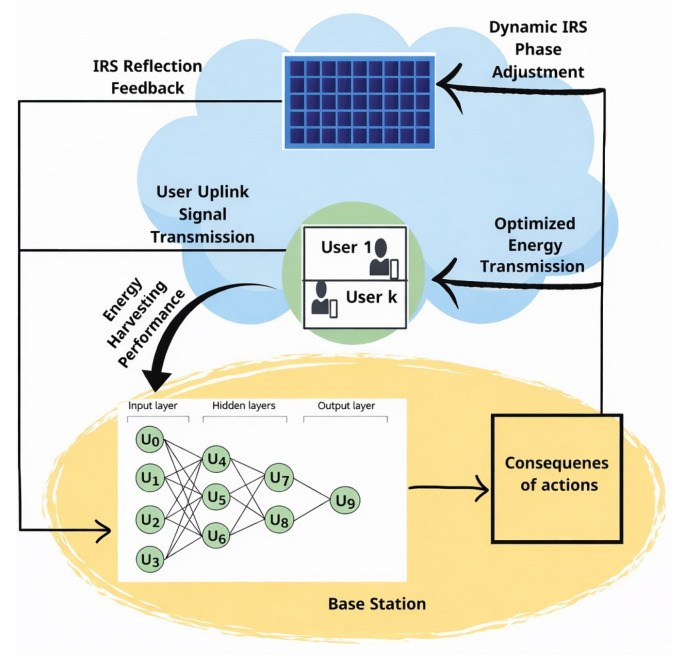
AI-driven IRS phase adjustment and uplink signal enhancement.

As seen in Figure 4, the DNN at the BS continuously optimizes the IRS phase shifts, which are then applied to maximize the desired metric (i.e., rate and energy). The system relies on real-time user feedback and environmental conditions to dynamically adjust the IRS phase shifts.

The process begins when users send uplink signals to the base station (BS). These uplink signals contain important information such as the energy harvesting status, channel conditions, and impact of previous phase shifts. This feedback is crucial for the DNN to determine whether sufficient energy is available and to evaluate the current propagation environment, thus allowing more precise IRS phase shift adjustments.

The DNN processes the input data and generates optimized IRS phase shifts. These shifts are then transmitted to the IRS, which reconfigures the reflecting surfaces to redirect the incoming signals toward the users. This enhances energy harvesting and ensures users receive the maximum possible harvested power.

While the IRS’s main role is to apply the new optimized phase shifts sent by the BS, it can also monitor the effectiveness of these adjustments. The IRS then sends reflection feedback to the BS to report key parameters, such as the reflected channel state, the effectiveness of phase shifts, and environmental variations. This feedback loop is essential because it allows the DNN to refine its predictions promptly to ensure that phase shifts remain optimized even when network conditions change.

#### 4.2.2. ML Model Complexity and Real-World Applicability

To further strengthen the proposed machine learning-based approach and address the computational limitations of traditional optimization methods, we analyze the computational complexity and performance accuracy of the DNN relative to conventional SDP methods. For the double IRS with $N_{1}=N_{2}=50$, the learning input dimension is

(18) $$d=1+N_{1}+N_{2}=101,$$

which corresponds to the transmit power and the two IRS phase vectors, while the network output is a scalar rate. Geometry-based parameters (distances and path loss) are fixed in our set-up and therefore are not included as explicit input features. The adopted MLP comprises three fully connected hidden layers with ReLU activations, each with [256,128,64] units. The total number of trainable parameters, denoted by $T_{p}$, is given by

(19) $$T_{p}=\left(d\cdot 256+256\right)+\left(256\cdot 128+128\right)+\left(128\cdot 64+64\right)+\left(64\cdot 1+1\right)=67329.$$

This corresponds to a memory footprint of approximately 0.26 MB using 32-bit floating point representation (or 0.52 MB using 64-bit representation). The inference complexity is dominated by the matrix-vector multiplications in the fully connected layers. Accordingly, the number of multiply–accumulate operations (MACs), denoted by $T_{\mathrm{MAC}}$, can be approximated as

(20) $$T_{\mathrm{MAC}}\approx d\cdot 256+256\cdot 128+128\cdot 64+64\cdot 1=66880.$$

Considering that one MAC corresponds to two floating-point operations (one multiplication and one addition), the total inference cost is approximately $2T_{\mathrm{MAC}}\approx 1.34\times 10^{5}$ FLOPs. In contrast, the SDP benchmark solved using CVX optimizes a Hermitian positive semidefinite matrix $X\in C^{n\times n}$ with $n=N_{1}+N_{2}=100$. A complex Hermitian matrix has $n^{2}$ real degrees of freedom, and hence the SDP contains $n^{2}=10,000$ real decision variables. In addition, it includes n=100 equality constraints from diag(X)=1 and a semidefinite cone constraint X⪰0 with a size of 100. Although storing the decision matrix *X* alone requires 100×100 complex entries (about 0.153 MB in double precision), interior point-based SDP solvers require substantially higher runtime and memory due to repeated matrix factorization and Newton iterations. This explains the clear computational gap between CVX-based optimization and the proposed MLP inference, consistent with the measured runtime results reported in Table 4.

Given the importance of computational efficiency in real-time IRS optimization, the DNN model was chosen for its practical deployment suitability and strong predictive performance. Therefore, although SDP provides a strong upper-bound benchmark, its runtime and resource requirements make it unsuitable for real-time operation.

This advantage stems from the fact that generating predictions with MLP primarily involves straightforward matrix operations and activation functions. In contrast, SDP relies on iterative solvers and requires substantial computational resources, whereas DNNs can produce accurate results almost instantly, making them ideal for real-time wireless applications.

## 5. Numerical Results

In this work, we intentionally centered our comparison on three primary benchmarks: SDP, random phase shift assignment, and a single IRS configuration. These were made to highlight established reference methods in the area. In particular, SDP is a classic standard in the literature for IRS optimization, as it provides a classical convex optimization method for reference when mathematical models of the optimization problem are perfectly ideal. On the other hand, the random phase scheme and single-IRS configurations are considered to provide lower-bound baselines and minimal implementation cases, as discussed in the literature [7,29].

The dataset was divided into three different training and testing configurations, namely (70%, 30%), (80%, 20%), and (60%, 40%). Ultimately, we considered the (80%, 20%) split set, as it yielded slightly better results than the other splits. To further verify that the proposed model generalizes well and does not overfit a specific split, we also performed a k-fold cross-validation procedure with k = 5. Scalability with respect to the IRS size was also assessed via training on a larger IRS dataset and testing on reduced phase dimensions (e.g., train at N = 70 and then test on a subset corresponding to N = 30). Table 5 reports each split configuration corresponding to its $R^{2}$ values for training and testing evaluations.

We positioned our user at the optimal location, (20,0) m, equidistant between both IRS panels located at (0,30) m and (0,−30) m. We assume that each IRS is equipped with 50 reflecting elements, i.e., $N_{1}=N_{2}=50$. Here, we set the path loss exponent to 2.8 for HAP-IRS links and 3.0 for IRS-user links. The power allocation factor for the near user was $\alpha_{\mathrm{near}}=0.4$, and for the far user, it was $\alpha_{\mathrm{far}}=0.6$. The path loss constant was set as $C=10^{-2}$.

### 5.1. Baseline Performance Under Perfect CSI (Ideal CSI)

**Figure 5:**
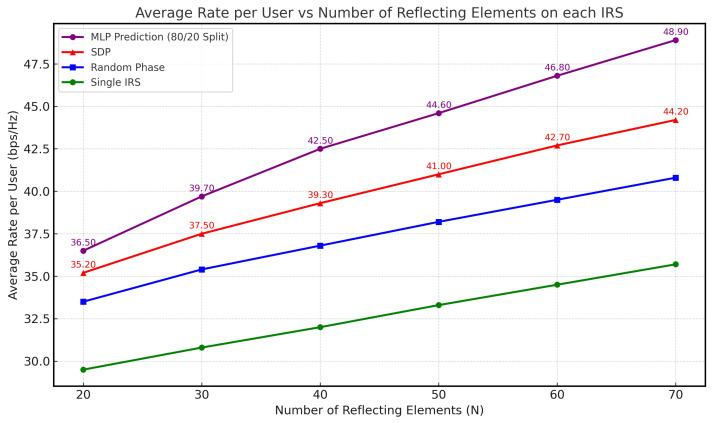
Average rate per user vs. number of IRS elements (N).

In this subsection, we present the baseline performance under perfect CSI, which serves as an upper-bound benchmark and helps isolate the benefits of the proposed architecture. Figure 5 illustrates the sum rate versus the number of reflecting elements (*N*) for each IRS. The results indicate that all cases exhibited an increasing trend as *N* grew, demonstrating that a higher number of reflecting elements enhances system performance. Our proposed deep neural network (DNN) approach demonstrated empirically competitive or superior performance relative to all benchmark methods, highlighting the strength of machine learning over traditional optimization techniques, especially for complex, high-dimensional optimization problems with non-convex constraints. In contrast, the random phase shift strategy and the single-IRS configuration yielded the lowest performance due to their limited signal enhancement capabilities and the absence of coherent beamforming.

**Figure 6:**
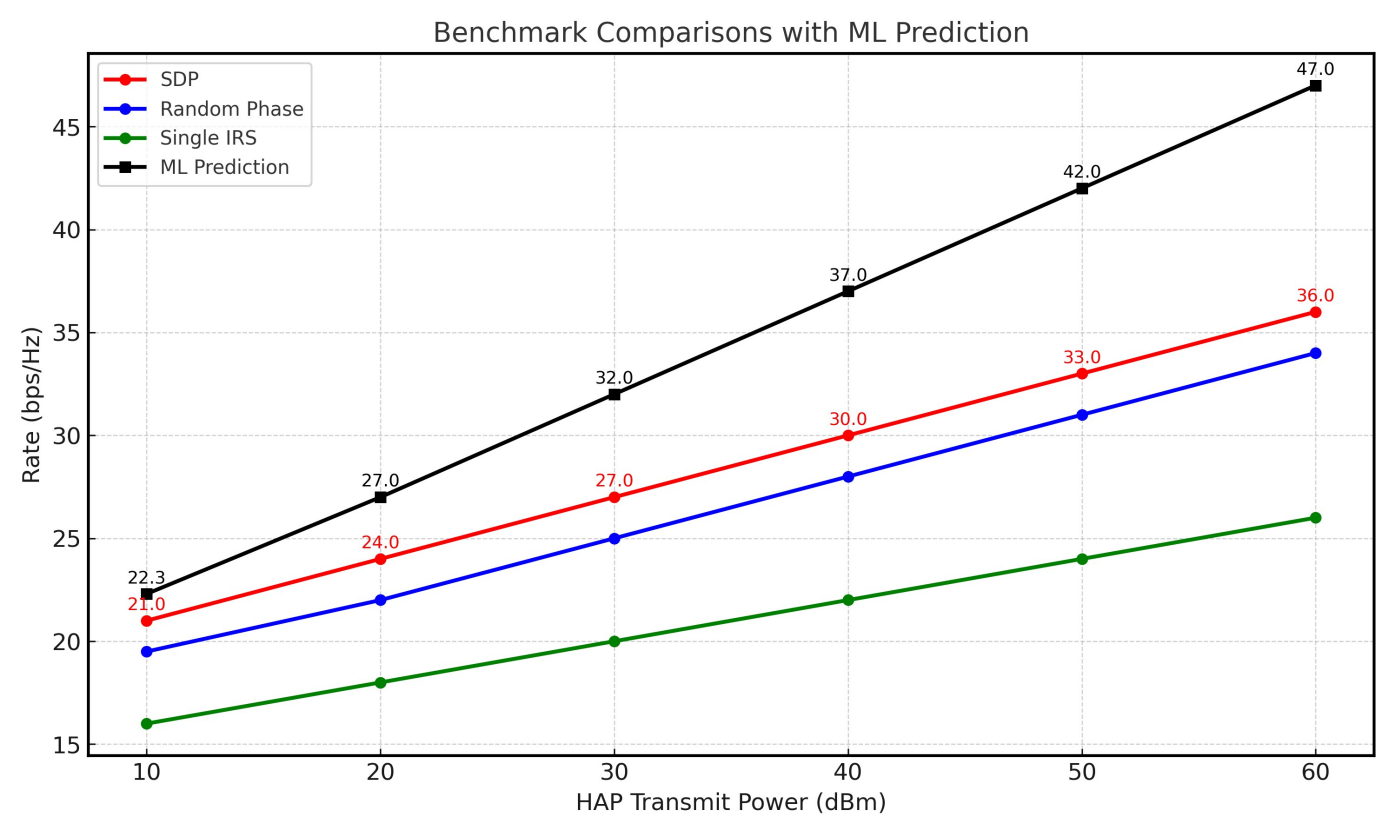
HAP transmit power vs. rate.

Figure 6 presents the sum rate versus the transmitted power ($P_{0}$) at the HAP across different scenarios. While all methods followed similar growth trends, the single-IRS case exhibited the slowest increasing rate, primarily due to the absence of the second IRS. Furthermore, our machine learning algorithm achieved the highest observed performance in this comparison, highlighting the critical role of IRS phase shift optimization in maximizing the sum rate.

The wireless energy transfer (WET) time needs to be optimized to ensure that users receive sufficient energy to sustain both uplink and downlink transmissions while enhancing overall system efficiency. Optimizing $\tau_{0}$ is particularly delicate, as it must balance energy transmission and data communication. If $\tau_{0}$ is too short, then users may harvest insufficient energy, leading to communication failures due to energy outages. Conversely, if $\tau_{0}$ is too long, then it reduces the available time for data transmission and significantly affects the achievable rate. For each channel realization and phase configuration, the script sweeps over candidate $\tau_{0}$ values (e.g., from 0.01 to 0.1) to find the value that maximizes the sum rate under energy harvesting and time-splitting constraints. The DNN predicts optimal phase shifts for a given $\tau_{0}$, while $\tau_{0}$ itself is optimized via the outer-loop grid search.

**Figure 7:**
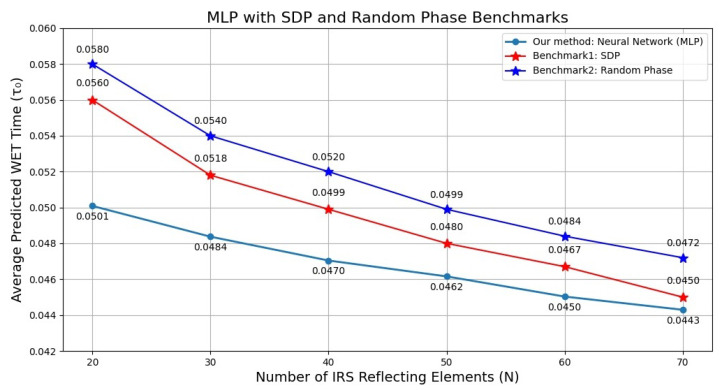
Average WET time vs. number of IRS elements (N).

Figure 7 shows the average predicted WET time $\tau_{0}$ with respect to the number of reflecting elements. As shown, interesting relationships between this parameter and system performance were discovered through neural network optimization. The optimal WET time $\tau_{0}$ decreased as the number of IRS reflecting elements increased. Specifically, when the system configuration had 20 elements, about 0.0501 time units were needed for energy harvesting, which decreased by approximately 11.6% to 0.0443 when 70 elements were used. This improvement is comparable to the SDP approach (which showed a 19.6% reduction from 0.056 to 0.045) and random phase allocation (which demonstrated an 18.9% reduction from 0.058 to approximately 0.047).

**Figure 8:**
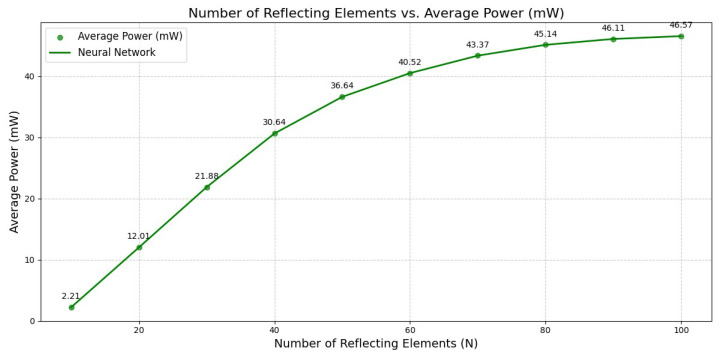
Number of reflecting elements vs. average harvested power.

Figure 8 shows the harvested power with respect to the number of reflecting elements. From the graph, we observe that at N=10, the average harvested power was only 2.21 mW, indicating minimal energy transfer due to the limited reflecting surface. However, the amount increased with the IRS size, reaching approximately 30.64 mW for 40 elements. This demonstrates a 14-fold improvement compared with the smallest configuration. This trend continued with larger IRS configurations; when N=100, the average harvested power reached 46.57 mW. This growth can be attributed to the array gain and the IRS’s enhanced capability to focus and reflect energy toward the users more efficiently. These results highlight the empirical effectiveness of increasing the number of IRS elements in improving wireless energy transfer.

**Figure 9:**
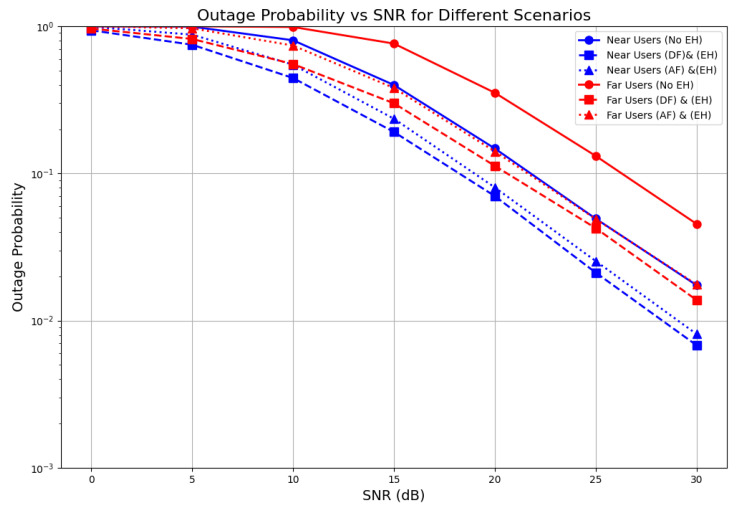
Outage probability across relaying schemes for near and far users.

Figure 9 shows the outage performance of the near user $U_{1}$ and the far user $U_{2}$ with respect to different values of the signal-to-noise ratio (SNR). Outage probabilities were evaluated using Monte Carlo simulations that exploited the system behavior under IRS phase shifts predicted by ML. The DNN does not directly estimate the outage. We investigated the outage probability of $U_{1}$ and $U_{2}$ under two different relaying schemes, namely decode and forward (DF) and amplify and forward (AF), considering both non-energy-harvesting and energy harvesting relaying schemes.

As we can see, the near users consistently performed better across all SNR values. With energy harvesting and DF relaying (blue dashed line), the near users achieved an outage probability of less than $10^{-2}$ at a 30 dB SNR. This significant improvement over the no EH scenario (blue solid line) demonstrates the effectiveness of our energy harvesting scheme.

The results show that while the AF relaying scheme with energy harvesting also provided substantial improvement over the no EH scenario, it lagged behind the DF relaying scheme at all SNR levels. For instance, at 30 dB, AF relaying achieved an outage probability slightly higher than $10^{-2}$, while the DF scheme dropped below this threshold.

On the other hand, the far users exhibited more interesting behavior in the results. Without energy harvesting (red solid line), they experienced higher outage probabilities, particularly at lower SNR values. However, their performance improved significantly with DF relaying and energy harvesting (red dashed line).

The graph shows that at a 30 dB SNR, the far users achieved an outage probability of about $10^{-2}$, representing a substantial improvement over the no EH case. The AF protocol (dotted lines) performed slightly worse than DF for both user types but was still better than the no EH scenario. This trade-off is expected, as AF is simpler to implement but also amplifies noise along with the signal.

### 5.2. Robustness Evaluation Under Imperfect CSI and Channel Aging

Phase shifts are usually designed based on the channel state information (CSI) in most practical IRS-assisted systems. However, CSI design is rarely perfect in real deployments and can be affected by estimation noise and feedback delay. In addition, user mobility and channel time variations may cause the CSI to become outdated even before the phase configuration is applied. These effects can reduce the gain of IRS beamforming and may impact the reliability of ML-driven phase design. To address this concern, we added a robustness study to assess whether the proposed ML-guided phase optimization remains effective under imperfect CSI. The main goal of this subsection is to verify that the ML approach is not only advantageous in ideal static conditions but also remains stable under more realistic scenarios. In particular, we considered two widely used robustness cases: (1) CSI uncertainty modeled as phase perturbation and (2) mobility or feedback delay modeled as channel aging. For a fair comparison, we evaluated the ML-guided design against strong benchmarks, including the real SDP solution (i.e., a CVX-based semidefinite relaxation solution used as a near-optimal benchmark for phase design, followed by phase extraction), a random-phase baseline, and a simplified single-IRS reference. The robustness results confirm that the ML-guided method followed the same general trend observed in the benchmark SDP solution. As expected, performance degraded when CSI became noisier or more outdated. However, the degradation remained controlled, and the ML-guided performance stayed close to SDP across the tested uncertainty and mobility levels. This provides strong evidence that the proposed learning-based phase optimization is not limited to the assumption of perfect CSI and is remarkably robust in practical IRS-assisted communications.

**Figure 10:**
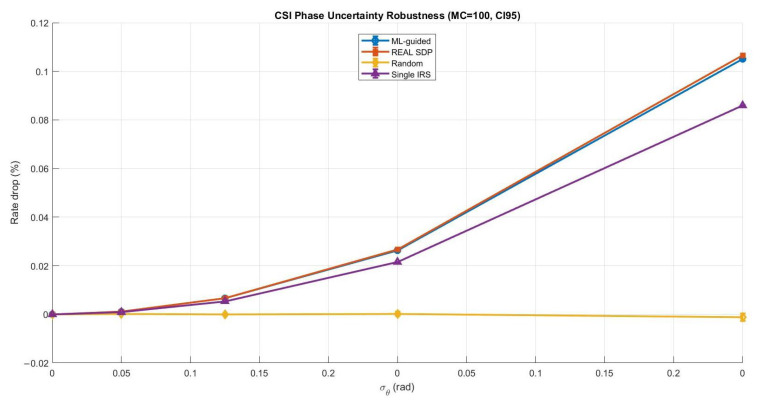
Comparing CSI phase uncertainty robustness (ML vs. benchmarks).

Figure 10 shows that both ML-guided and real SDP remained highly stable under CSI phase noise, with only a rather small rate loss even at the largest error level. In contrast, the random phase baseline exhibited a larger drop and wider variability, which confirms its sensitivity to CSI mismatch and uncontrolled phase configurations. The single-IRS baseline also suffered from higher degradation than the double-IRS solutions, since disabling one IRS reduced the available degrees of freedom for compensating uncertainty.

**Figure 11:**
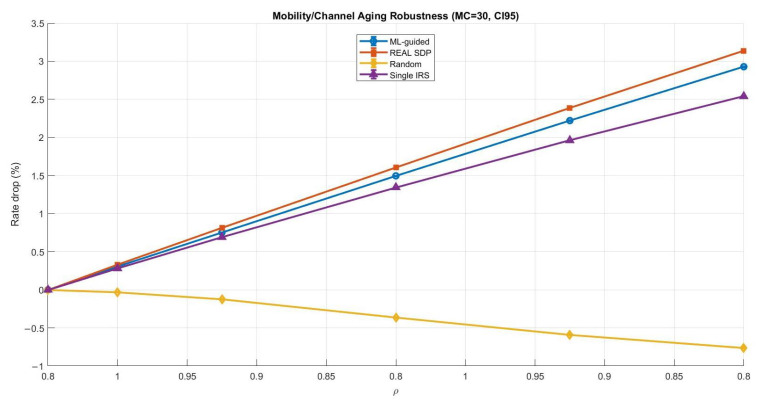
Comparing Mobility and channel aging robustness (ML-guided vs. benchmarks).

Figure 11 shows a clearer degradation trend as ρ decreased, which means that the mobility had a stronger impact than phase uncertainty. Overall, the ML-guided method still tracked the SDP benchmark closely. Compared with ML-guided and real SDP, the single-IRS baseline exhibited greater degradation, whereas the random phase strategy yielded the weakest robustness and the least predictable performance.

In this robustness evaluation, the selected CSI phase uncertainty levels were $\sigma_{\theta }=\left[0,0.02,0.05,0.10,0.20\right]$ rad to represent a practical range from mild estimation errors to clearly imperfect CSI conditions, where $\sigma_{\theta }=0.20$ rad corresponds to a severe phase distortion case. Similarly, the mobility and channel aging factor was modeled using ρ=[1,0.98,0.95,0.90,0.85,0.80] to cover scenarios from quasi-static channels (ρ=1) to faster channel variations and delayed feedback (ρ=0.8). This selection allowed a controlled and meaningful evaluation of robustness under realistic CSI degradation and mobility conditions. To ensure that the reported robustness curves were quite reliable statistically and not affected by random perturbation realizations, we applied Monte Carlo (MC) averaging. We used MC =100 for CSI phase uncertainty since it had a relatively small impact, and therefore, more trials were required to obtain smooth and reliable estimates. For the mobility and channel aging case, MC =30 was sufficient because the degradation trend was stronger and more consistent across realizations. Moreover, 95% confidence intervals (CI95) are shown as error bars to explicitly quantify variability and statistical confidence in the rate degradation results.

## 6. Conclusions

To enhance the quality of the received signal in scenarios with limited line of sight (LoS), we propose a double IRS-assisted C-NOMA system that leverages wireless energy transfer.

The key contribution is to use a machine learning model to optimize the double-IRS phase shifts without relying on supervised labels from optimization solvers. We demonstrated that this learning-based approach achieves comparable or superior performance in terms of outage, harvested power, and time allocation at significantly lower complexity. The analysis captured the system’s joint behavior under practical energy constraints and relay protocols (DF and AF), an aspect that has not been addressed in this form before. These insights provide useful design directions for 6G systems in smart environments, particularly for low-power IoT and remote-access networks. This paper considered a perfect SIC for ease of analysis and to benchmark the attainable performance. To improve practical relevance, we further evaluated the proposed ML-guided IRS design under imperfect CSI conditions, i.e., CSI phase uncertainty and mobility or channel aging. In future work, we plan to explore other state-of-the-art ML models, such as deep reinforcement learning (DRL) and graph neural networks (GNNs), and incorporate practical impairments, such as residual interference and phase quantization, to better study and generalize these findings.

## Figures and Tables

**Table 1:** Key system model parameters.

Parameter	Value(s)
Transmit power ($P_{\mathrm{number}}$)	[10, 20, 30, 40, 50, 60] dBm
Bandwidth (BW)	1 MHz
Number of users ($U_{n}$)	2
Path loss constant (*c*)	$1\times 10^{-2}$
Phase shifts randomness	Uniform in [−π,π]
Randomness factor	0.3

**Table 2:** ML model configurations.

Model	Parameter	Value
MLP	hidden_layer_sizes	(256, 128, 64)
	max_iter	1000
	random_state	42
KNN	n_neighbors	5
SVR	kernel	rbf
	epsilon	0.1
Random Forest	n_estimators	100
	random_state	42

**Table 3:** Performance evaluation of the tested ML regression models.

Model	MSE	RMSE	MAE	NRMSE	$R^{2}$
Random Forest	1.190	1.090	0.832	0.020	0.993
MLP	2.751	1.579	1.051	0.036	0.989
SVR	6.620	2.573	1.801	0.047	0.906
KNN	20.601	4.538	3.512	0.083	0.708

**Table 4:** Comparison of average runtime per sample between SDP and DNN inference.

Method	Platform	Avg. Runtime/Sample (s)
SDP (CVX)	Python 3.10.0 (Intel i7, 16 GB RAM)	2.87
DNN inference	Python (TensorFlow 2.12.0, CPU)	0.1688

**Table 5:** Train and test $R^{2}$ values for different dataset split configurations.

Split Configuration	Train $R^{2}$	Test $R^{2}$
80/20	0.9896	0.9835
70/30	0.9890	0.9816
60/40	0.9874	0.9690
Train N=70/Test N=30	0.9865	0.9585

## Data Availability

The data presented in this study are available on request from the corresponding author. The data are not publicly available due to their use exclusively for academic research and their storage in a local environment.
